# A New and Instructive Study by Dr. Brownlee

**Published:** 1918-11-30

**Authors:** 


### EPIDEMIOLOGY OF PULMONARY TUBERCULOSIS.

### A New and Instructive Study by Dr. Brownlee.

A new and instructive study on the epidemiology of pulmonary tuberculosis has recently been published under the auspices of the Medical Research Committee. The author is Dr. Brownlee, Director of the Statistical Department of the Committee, and a study of its pages yields evidence of the careful manner in which the investigation has been carried out. It is not proposed in reviewing this report to give any lengthened description of the data upon which it is based, or the manner in which the statistical inquiries have been carried out, but rather to discuss briefly the conclusions at which Dr. Brownlee has arrived. The most striking feature of the investigation is that as a result of a statistical investigation certain conjectures as to the existence of different strains of the tubercle bacillus have been arrived at.

Dr. Brownlee selected as his data the deaths registered as due to pulmonary tuberculosis since the introduction of the registration of deaths in 1856, and also certain previous data. The conclusion that Dr. Brownlee arrived at as a result of his initial inquiry, published in *Public Health* in June 1918, was that pulmonary tuberculosis is a disease of several types distinguished by their age-incidence. There are three ages at which tuberculosis of the lungs most frequently causes death—namely, 20-25, 45-50, and 55-65—and these three types are described as having different geographical distribution in the British Isles. In the introduction to the report the statement is made that the results of clinical observation as such have not yet pointed to any separation of lung tuberculosis into distinct types attacking life at different ages. This statement we are not prepared to accept in its entirety. As a result of clinical observation pulmonary tuberculosis has been divided into three types, according to variations presented at different ages; namely, the type of childhood, the adult type, and the senile type. Root and pulmonary tuberculosis affecting young children under the age of fifteen is a distinctive type by itself, but as it is relatively non-fatal, no conclusions regarding it can be drawn from the data based on the death returns. The importance of the latent, non-fatal type in children lies in its possible ætiological relationship to the development of one or other of the three subsequent types to which Dr. Brownlee refers.

That the author's investigations have led him to the conclusion that pulmonary tuberculosis is not a single disease but rather a group of diseases is an interesting fact in accordance with the results of clinical observation as to the extreme variation in type which this disease presents, not only in different age groups, but in the same age group. The variation in the clinical picture presented by the young adult type has not infrequently been referred to. There is also considerable clinical evidence in favour of the theory that the middle-age and senile type is not usually a fresh infection, but rather a recrudescence of the young adult type. Further information is necessary with regard to the factors which determine the fatal termination of this young type or its prolongation into later years. Dr. Brownlee's conclusions as to the ætiological factors which bear on the earlier and later types are interesting, although they rather upset previous views. He states that it would seem that the middle-age type of pulmonary tuberculosis is an infection the virulence of which is increased by the presence of unhealthy conditions, whereas the spread of the young adult and senile type is not affected by such conditions. He points out the possibility that when a large number of persons die from this disease between the ages of 20-25 the number of survivors in the population at the age of 30-50 who are susceptible to infection may be so few as to limit the possibility of any great number of cases at these ages. He further states, however, that it is equally possible and equally probable that wide special infection in youth may induce an active immunity which protects them for many subsequent years or even for the whole of life. Lastly, Dr. Brownlee refers to the geographical distribution of the young adult and middle-age types, and finds that whereas the former type is more frequently met with in the coast districts, the latter type is more prevalent in the urban districts from London to the Northern Midlands. The experience of the present war does not support the view that the young adult type of pulmonary tuberculosis is not affected to any appreciable extent by environment. Since the year 1914 there has been a steady increase in the incidence of this type of tuberculosis of the lungs in the military and civil population—an increase which can only be put down to adverse environmental conditions, while the type itself has shown increased virulence and severity. The suppositions that pulmonary tuberculosis is not a single disease but rather a group of diseases, and that there exist different strains of the tubercle bacillus, raise some interesting points for consideration in relation to treatment. Dr. Brownlee refers to the necessity in vaccinetherapy to see that the vaccine is made from the proper organisms. He claims that the use of tuberculins made from the wrong strains of tubercle baccilli may be partly responsible for the different views held by physicians as to the real value of tuberculin in the treatment of lung tuberculosis, and states that he never saw any demonstrable benefit from its use in Glasgow. He suggests as a probable explanation of this that whereas there is little of the middle-age type of pulmonary tuberculosis in Glasgow, the tuberculins used were no doubt chiefly made from the tubercle bacilli of this type which is the prevalent type in London. This supposition is, at any rate, in agreement with the views held by the majority of bacteriologists that in vaccinetherapy an autogenous

**Epidemiology of Pulmonary Tuberculosis**—(*cont.*)

vaccine is the one calculated to give the best results; a claim, however, with stronger theoretical than practical support. Mention must be made of two further points referred to in the report. Dr. Brownlee describes the epidemiology of the young adult and the middle-age types as different; and states that while the present epidemic of the former type had its maximum about the middle of last century, the epidemic of the middle-age type had its maximum fully one hundred years ago. His final point is that the alteration which has taken place in the mean age at which death from pulmonary tuberculosis occurs, an alteration which is characterised by a steady rise, would, on the face of it, appear to be due to increased immunity; such a rise would necessarily follow if the young adult type is the type of the disease which is disappearing most rapidly. Dr. Brownlee proposes to continue his inquiry, and in the meantime he is to be congratulated on a commendable piece of work on an important subject.

